# Robotic-assisted differential total knee arthroplasty with patient-specific implants: surgical techniques and preliminary results

**DOI:** 10.1186/s42836-024-00255-1

**Published:** 2024-06-10

**Authors:** Hanlong Zheng, Mingxue Chen, Dejin Yang, Hongyi Shao, Yixin Zhou

**Affiliations:** Department of Orthopaedic Surgery, Beijing Jishuitan Hospital, Capital Medical University, No. 31, Xinjiekou East Street, Xicheng District, Beijing, 100035 China

**Keywords:** Total knee arthroplasty, Patient-specific, Customized, Soft-tissue, Gap balancing

## Abstract

**Objective:**

In total knee arthroplasty (TKA), achieving soft-tissue balance while retaining acceptable lower limb alignment is sometimes difficult and may lead to patient dissatisfaction. Theoretically, patient-specific implants can bring great benefits, while the lack of precise surgical tools may hinder the improvement of outcomes. The objective of this study was to illustrate surgical techniques and evaluate kinematics and early clinical outcomes of robotic-assisted TKA using patient-specific implants.

**Methods:**

Based on preoperative CT scan, femoral and tibial components were 3D printed. Medial and lateral tibial liners were separate with different thicknesses, posterior slopes and conformity. TiRobot Recon Robot was used for surgery, and was armed with smart tools that quantify gap, force and femoral-tibial track. We collected data on demographics, intraoperative gap balance and femoral-tibial motion. In the follow-up, we evaluated the range of motion, Visual Analogue Scale (VAS), forgotten joint score (FJS), Knee injury and Osteoarthritis Outcome Score, Joint Replacement (KOOS, JR) score. Radiological data were also harvested.

**Results:**

Fifteen patients (17 knees) were enrolled with a mean age of 64.6 ± 6.4 (53–76) years. In 5 knees, we used symmetric tibial liners, the rest were asymmetric. After surgery, the average alignment was 1.6 ± 2.0 (-3–5) degrees varus. The average follow-up lasted 6.7 ± 4.2 (1–14) months. The mean visual analogue scale was 0.8 ± 0.7 (0–2), FJS was 62.4 ± 25.3 (0–87), KOOS was 86.5 ± 9.4 (57–97). 11 patients were “very satisfied”, 3 were “satisfied" with the result, and one patient was neutral due to restricted extension and unsatisfactory rehabilitation at five months’ follow-up.

**Conclusions:**

With patient-specific implants and robotics, TKA could be performed by a mathematical way, which was dubbed a “differential” TKA. Intraoperative kinematics was excellent in terms of gap-force balancing and femoral-tibial relative motion. Preliminary clinical outcomes were overall satisfactory.

**Supplementary Information:**

The online version contains supplementary material available at 10.1186/s42836-024-00255-1.

## Introduction

Over the past decades, total knee arthroplasty (TKA) has been proven to be an effective treatment for end-stage osteoarthritis, achieving satisfactory pain relief and deformity correction. However, 15%–20% of patients are still dissatisfied with the result, complaining of persistent pain, stiffness or instability [1, 2]. The difficulty in achieving soft-tissue balancing while retaining acceptable lower limb alignment is one of the major reasons for postoperative dissatisfaction or surgical failure [3].

Merchandized TKA implants have gone through several generations and come in a great many designs. Although their geometry design could universally adapt to most patients, the contradiction between the single design of surface geometry and diverse individual bony anatomy/soft-tissue characteristics could not be neglected [4], and can sometimes lead to overhang, notching and abnormal kinematics. In the past decade, while patient-specific implants and instruments [5] have been reported to yield better clinical outcomes [6–8], have less adverse events [9], and attain more accurate implant fit and leg alignment [10] and better kinematics [11], some authors believed that patient-specific implants are no different as compared to off-the-shelf products in terms of clinical outcomes [12, 13]. Currently, the most widely reported customized TKA is iTotal CR (Conformis, Inc., Billerica, MA, USA) [5]. On the basis of preoperative CT scan, the shape of implants could well fit the original anatomy of the knee, and patient-specific instruments (PSI) could also be used for accomplishing accurate bone cutting [14].

Despite some theoretical advantages, the limitations of current patient-specific implants are obvious [15]. First, positioning the implants accurately could not be well achieved with PSI [15]. More than 1-mm bone-cut deviation could affect ligament tension and gap-balancing. Second, joint gap, ligament tension and femoral-tibial motion could not be intraoperatively quantified.

With advances in robotic technology, robotic-assisted TKA could help surgeons precisely perform bone cutting, control alignment and implant positioning [16, 17]. To our knowledge, no authors have reported the combination of customized TKA and robotic surgery. Using TiRobot Recon Robot (TINAVI, Beijing, China), customized implants, and surgical tools that quantify gap, force and ligament balancing, surgeons could modify gaps at a 1-mm accuracy across the full range of motion, while keeping reasonable alignment. This procedure is called “differential” TKA, named after a mathematical term. Our prospective study aimed to illustrate surgical techniques, to evaluate kinematics and early clinical outcomes of robotic-assisted differential TKA with patient-specific implants.

## Patients and methods

### Patient selection and implant customization

This cohort study was approved by the institutional review board (IRB) of our institute. The inclusion criteria were:

1. Patients agreed to receive customized TKA.
2. Patients at the age between 50 and 80 years.
3. Patients with varus or flexion deformity less than 20 degrees, and valgus deformity less than 15 degrees.

The exclusion criteria were:

1. Any instability of the knee, or posterior cruciate ligament (PCL) deficiency.
2. Patients who refused to receive postoperative follow-up.
3. Patients whose intraoperative data were not available.

A total of 15 patients (17 knees involved) were enrolled.

**Fig. 1:**
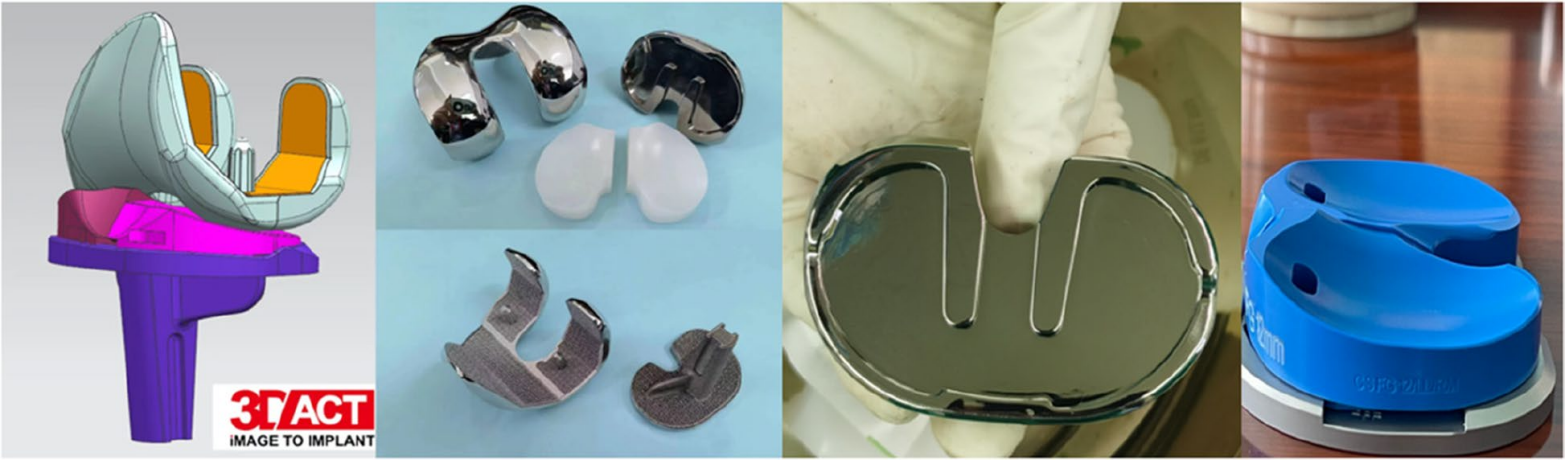
3D Printed patient-specific TKA components and trials

At the outpatient clinic, patients routinely received a CT scan of the affected limb, including the femoral and talus center, and then the engineer modified the contour of femoral condyle and the tibia plateau. Through physical examination (posterior drawer test) and femoral-tibial relationship in lateral view of X-ray, PCL function was evaluated. The design of femoral component originates from off-the-shelf product of A3 GT (AK Medical, Beijing, China) with cruciate-retaining (CR) design. Femoral size depended on the antero-posterior dimension, and component width was adjusted in accordance with femoral anatomy. The width of the anterior flange was optimized in accordance to anatomy. Geometry of tibia component derived from off-the-shelf product of TMK (AK Medical, Beijing, China), with minor adjustments made to achieve the best tibial coverage. Medial and lateral tibial liners were separate (shown in Fig. 1), ranging from 8 to 14 mm, increasing by 1 mm in thickness. 3 or 6 degrees of posterior slope was chosen, and the manufacturer also provided a deep-dish design of tibial inserts, with more restriction in flexion and roll-back. The procedure of 3D printing and processing took six weeks before operation. Based on CT scan, the robotic system TiRobot Recon, produced by an orthopedic robot enterprise in China, was compatible with implants of different manufacturers.

### Surgical procedures

#### Step 1

All the procedures were performed by the same senior surgeon (corresponding author Yixin Zhou). We routinely made an anterior mid-line longitudinal incision via the medial parapatellar approach. After removal of osteophytes, ACL was resected, and PCL integrity and tension were re-checked to make sure that a CR patient-specific TKA could be performed. After bone registration, we routinely applied a varus/valgus stress in extension and 90 degrees of flexion, to evaluate the laxity of medial and lateral soft tissues, and then adjusted component position for pre-resection balancing. This step is illustrated by Video S1 (Supplementary Materials).

#### Step 2

**Fig. 2:**
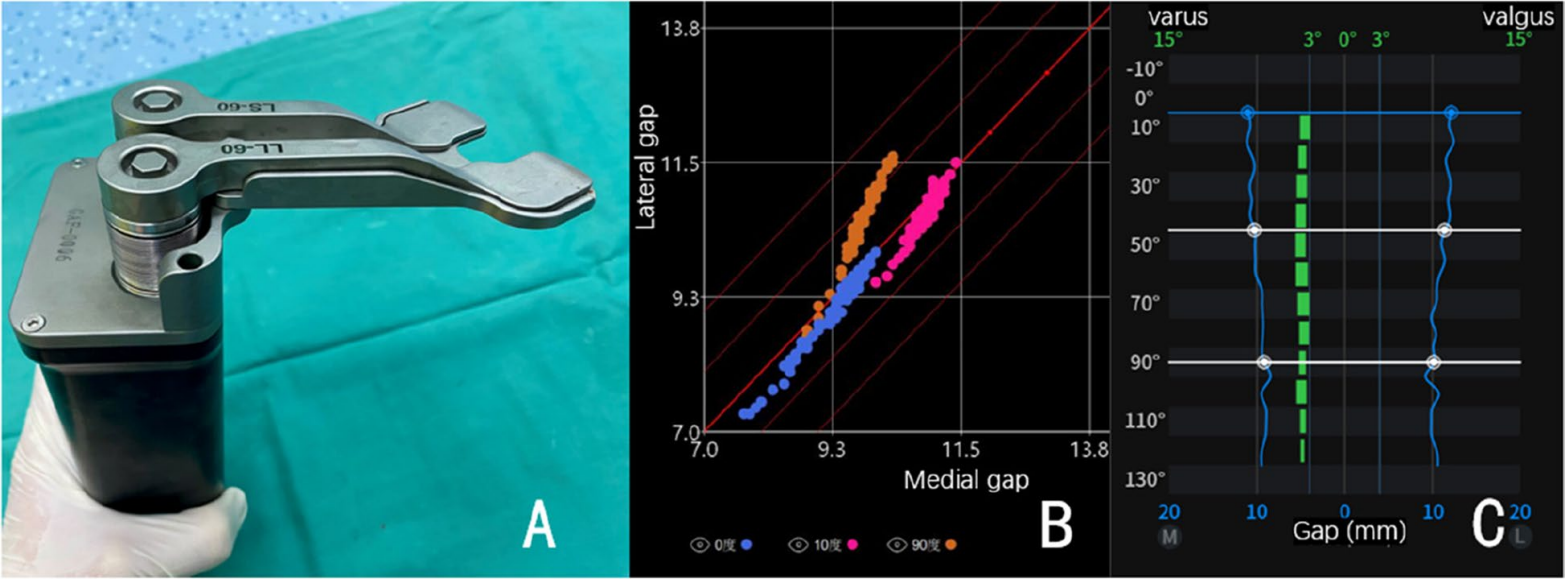
Smart tools for gap balancing and kinematics. **A** Soft-tissue balancing Solver. **B** Matrix formed by the Solver. Blue points were 0° of extension, pink 10° of extension, and orange 90° of flexion. **C** When the Solver exerts a symmetric force, medial and lateral gap variance is indicated by a blue line

Using sequential bone cutting (SBC) technique, as we previously reported [18], distal femur, posterior chamfer femur, posterior femoral condyle and the tibia were cut by using bone saw through the slot of the robotic arm. Anterior femoral condyle and anterior chamfer femur were reserved for potential adjustments. After creating an initial flexion and extension gap, we inserted a pre-cutting trial of the femur. This trial had a surface geometry identical to the AK femoral condyle and was 2-mm thinner without anterior flange. We used an innovative smart tool dubbed Solver (TINAVI, Beijing, China), to quantify medial lateral gaps and collateral ligament elasticity (Fig. 2). By applying stepwise tension on the medial and lateral lever between the pre-cut femoral trial and the tibia bone-cut surface, the Solver formed a gap-force matrix that can guide the bone cutting. In extension (10°), mid-flexion and flexion, as highlighted in different colors, the matrix could show the elasticity of collateral ligaments, the absolute value of medial and lateral gaps, and medial–lateral gap difference at each angle of flexion. By analyzing the Solver matrix, surgeons could make minor changes in bone cutting. If balance could not be achieved by bone cutting, soft tissues could be released until the matrix of different colors got well-overlapped. This step is illustrated by Video S2 (Supplementary Materials).

#### Step 3

**Fig. 3:**
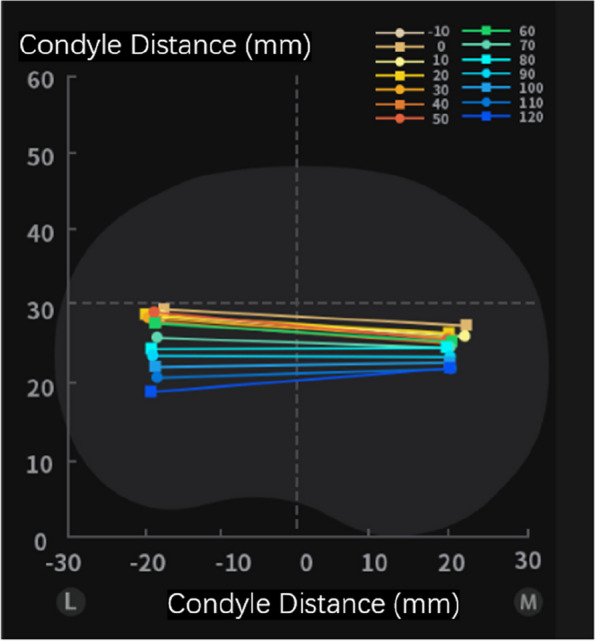
Femoral-tibial track formed by the TiRobot Recon Robot. Femoral-tibial contact point at each degree of flexion is highlighted by different colors. L means lateral and M medial

With satisfactory bone-cutting, anterior femoral condyle and anterior chamfer femur was resected. Tibial and femoral trials were inserted, with separate tibial liners. If the bone-cutting and Solver matrix were in line with the lower limb mechanical axis, medial and lateral liners of different thicknesses could be adopted. Afterwards, the TiRobot Recon Robot would capture the femoral-tibial contact points through full range of motion, presenting the pattern of femoral roll-back and mid-flexion stability (Fig. 3). This step is shown in Video S3 (Supplementary Materials). If the femoral-tibial track was satisfactory, tibial preparation and prostheses implantation could be proceeded. The surgeon could also try deep-dish tibial liner for more stability, as illustrated in Video S4 (Supplementary Materials).

### Postoperative management and data collection

Demographics were routinely recorded. During operation, we collected following data: operation time, medial and lateral gap in extension and 90° flexion measured by the robot, component size, tibial liner type and thickness, femoral and tibial roll-back. Gap was defined as the vertical distance from the medial/lateral condyle to the tibial bone-cut surface upon assembly of the femoral component trial. Any soft-tissue release was recorded. After operation, patients were given the same rehabilitation instructions as in routine TKA. Three days after operation, we evaluated the result on Visual Analogue Scale (VAS) and the flexion of the affected knee. In the follow-up at the out-patient clinic, we collected such data as the range of motion, VAS scores, forgotten joint scores (FJS), Knee Injury and Osteoarthritis Outcome Score, Joint Replacement (KOOS, JR), and satisfaction as patient-reported outcomes (PROMs). For radiological evaluation, we measured the lower limb alignment and tibial varus angle.

## Results

Of the 15 patients (17 knees) who underwent customized TKA, 8 were female and 7 were male, with a mean age of 64.6 ± 6.4 (53–76) years and a mean body mass index (BMI) of 30.0 ± 4.2 (23.7–37.6). The average alignment before operation was 6.0° ± 7.6° (-12–18) varus, including 2 valgus knees.

Intraoperatively, the mean medial extension gap was 11.1 ± 1.0 (9.1–12.7), lateral extension gap was 11.5 ± 0.9 (9.9–12.7), and in 90° flexion, the mean medial gap was 10.2 ± 1.2 (8.8–12.5), and lateral gap was 11.2 ± 1.3 (8.9–13.7). In 5 knees, we used symmetric tibial liners. Eight knees used liners of different thickness, with the difference being within 1 mm in all cases, and 4 knees used liners of the same thickness but of different tibial slopes. Deep-dish plates were applied to 4 knees, all on the medial side. Two patients underwent soft tissue release due to severe deformity. The mean medial roll-back was 9.1 ± 2.1 (6–12) mm, and lateral roll-back was 13.2 ± 3.1 (10–19) mm. The mean time of operation lasted for 99.4 ± 20.5 (60–130) minutes.

After surgery, the average alignment was 1.6 ± 2.0 (-3–5) degrees varus, and the mean tibial varus angle was 1.4 ± 0.9 (-1–2.5) degrees. Two days after operation, the average VAS was 2.5 ± 1.0 (2–5) and the mean flexion was 86.7 ± 7.9 (70–100). The average follow-up was 9.1 ± 4.1 (4–17) months. No patient required further operation, and no infection was observed. One patient developed deep venous thrombus, which was addressed by conservative treatment and immobilization. Another patient underwent lumbar surgery after TKA. At the most recent follow-up, the mean VAS was 0.8 ± 0.7 (0–2), FJS 62.4 ± 25.3 (0–87), and KOOS 86.5 ± 9.4 (57–97). The average range of motion was 1.7 ± 3.5 (0–10) degrees of extension, and 119.7 ± 12.8 (95–140) degrees of flexion. 11 patients were “very satisfied” with their operation results (including two bilateral cases), 3 patients were “satisfied”. One patient was “neutral” due to restricted extension and unsatisfactory rehabilitation at five months follow-up.

## Discussions

TKA has reached its ceiling effect after two decades. Although the overall satisfaction is high, it seems difficult to satisfy the remaining 10 to 20% “unhappy” patients [19]. Achieving both soft-tissue balance and alignment is sometimes challenging, and this paradox may hinder the TKA result from going further to “very good” from “good”. With use of robotic technology and accurate bone-cutting, achieving gap balance while keeping acceptable alignment is feasible in most cases. However, adjusting bone-cut by quantifying soft tissue gap and tensions remains a difficult task [16, 17], because soft-tissue elastic modulus could be patient-specific. Moreover, the mismatch between anteroposterior (AP) dimension and mediolateral (ML) width is another problem, as Asian knees sometimes present as a narrow shape, with larger AP and smaller ML, resulting in overhang, notching or more flexion of the femoral component [2]. Optimizing patient-specific prosthesis design could be an effective solution to tailor each type to individual bony anatomy and soft-tissue characteristics.

Our study showed excellent balance and kinematics during robotic-assisted patient-specific TKA, as most flexion and extension gaps were balanced within 1 to 2 mm, and femoral-tibial track was smooth. In cases of “asymmetric” gaps, either medial–lateral or flexion–extension imbalanced, asymmetric tibial trays could be applied, to avoid excessive soft-tissue release or additional bone-cut, which may cause further dynamic imbalance and mid-flexion instability. The general strategy was: if the extension gap was balanced while tight in medial flexion, which is the most common gap balance mode in CR TKA, a liner of the same thickness but with more posterior slope could be adopted; if the medial gap was tight in both extension and flexion, which is another common scenario, a medial liner of thinner thickness could be used; if the extension and flexion gaps are individually balanced while flexion is more narrow than extension, we chose symmetric tibial liners with more posterior flexion. Deep-dish designs were used for cases with slightly larger medial extension gap (less than 1 mm) or with excessive medial roll-back. The aforementioned concepts of “differential” TKA could be explained in mathematical terms, which helps surgeons attain optimal gap balance, i.e., within 1 mm.

Our research showed an overall satisfaction at short-term follow-up. Previous studies comparing patient-specific implants and off-the-shelf implants yielded varying outcomes. Some authors believed that patient-specific implants are no superior or even inferior to their non-patient-specific counterparts, in terms of functional scores or the range of movement, when compared to off-the-shelf implants [20]. Small sample size, heterogeneity and inaccurate bone-cut could be ascribed to such different results. Recently, one of the most convincing studies by Schroeder et al. [7] compared early clinical outcomes and implant preference in 47 patients (94 knees) undergoing staged bilateral TKA, all patients had one knee receiving patient-specific implant (iTotal) and the other knee having off-the-shelf implant. 72.3% of the patients preferred patient-specific implants, 21.3% felt no difference and 6.4% had preference for off-the-shelf implants. With our current study, some patients (although expressed satisfaction) are still complaining of some swelling, pain, or stiffness, especially those who were followed up for less than 6 months, indicating that good intraoperative kinematics does not always lead to fast recovery or excellent clinical outcomes. Rehabilitation period could probably be a confounding factor, and long-term follow-up are inevitably required.

In terms of radiological outcomes, we used robots to control tibial varus to a maximum of 4 degrees, while respecting mechanical alignments. Previous studies showed that the alignments of customized TKAs were closer to neutral status, with fewer outliers of 3 degrees [12, 21], while other studies found no significant difference [12]. In our study, two cases were outliers of alignment (4 and 5 degrees, both with severe varus deformity), as the surgeon planned by using the robot. With accurate alignment control, robotics provide more options of alignment types [22] that surgeons choose from. With separate designs of tibial liners, 1-mm thickness difference (usually medial side thinner than lateral one) leads to only 1 degree of alignment change, which is usually acceptable. With asymmetric tibial plates, varus of tibial component could be decreased, which may potentially improve long-term survivorship [22].

This study has several limitations. First, the sample size was small, and follow-up time was short. To our knowledge, no previous study reported robotic-assisted TKA with patient-specific implants. Moreover, this was also the first report to use smart tools to quantify gap-force balance and the pattern of femoral-tibial relative motion. The goal of our study was not only to report short-term clinical effects of this surgical procedure, but more importantly, to initiate a “differential” method that integrate gap, force, and alignment, aiming for more physiological kinematics and maximal patient satisfaction. Second, there were no control groups to illustrate the advantage of robotic-assisted TKA with customized implants over manual TKA or regular robotic TKA. Nevertheless, future research efforts involving larger sample sizes and control groups are needed to report on the mid- to long-term results. Moreover, basic studies [23–26] are also warranted to clarify the relationship between bone-cut, joint gap, ligament tension, alignment, implant geometry, tibia plate conformity, among others. To achieve more individualized implant design, geometry of femoral condyle curve ratio can also be optimized to patient-specific anatomy and soft issue characteristics.

## Conclusions

In conclusion, robotic-assisted TKA with patient-specific implants showed excellent intraoperative kinematics in terms of gap-force balancing and femoral-tibial relative motion. Early clinical and radiological outcomes were overall satisfactory. Future studies with larger sample sizes and longer follow-up are needed, to compare the mid- to long-term outcomes of traditional manual TKA, robotic-assisted TKA with off-the-shelf implants, and robotic-assisted TKA with patient-specific implants. Basic studies are also required to clarify the relationship between implant geometry, bone-cut, gap balancing and kinematics.

### Supplementary Information

Supplementary Material 1. Video S1. Exposure, Registration and Pre-section Balancing.

Supplementary Material 2. Video S2. Initial Bone Cutting and Solver Test.

Supplementary Material 3. Video S3. Secondary bone cutting and femoral-tibial track test.

Supplementary Material 4. Video S4. Deep-dish medial liner vs. standard medial liner, and final implantation.

## Data Availability

The datasets used and/or analysed during the current study are available from the corresponding author on reasonable request.
